# Vaccine effectiveness against SARS-CoV-2 transmission and infections among household and other close contacts of confirmed cases, the Netherlands, February to May 2021

**DOI:** 10.2807/1560-7917.ES.2021.26.31.2100640

**Published:** 2021-08-05

**Authors:** Brechje de Gier, Stijn Andeweg, Rosa Joosten, Ronald ter Schegget, Naomi Smorenburg, Jan van de Kassteele, RIVM COVID-19 surveillance and epidemiology team, Susan JM Hahné, Susan van den Hof, Hester E de Melker, Mirjam J Knol, Members of the RIVM COVID-19 surveillance and epidemiology team, Members of the RIVM COVID-19 surveillance and epidemiology team, Agnetha Hofhuis, Anne Teirlinck, Alies van Lier, Bronke Boudewijns, Carolien Verstraten, Guido Willekens, Irene Veldhuijzen, Jan Polman, Jan van de Kassteele, Jeroen Alblas, Janneke van Heereveld, Janneke Heijne, Kirsten Bulsink, Lieke Wielders, Liselotte van Asten, Liz Jenniskens, Loes Soetens, Maarten Mulder, Maarten Schipper, Marit de Lange, Naomi Smorenburg, Nienke Neppelenbroek, Patrick van den Berg, Priscila de Oliveira Bressane Lima, Rolina van Gaalen, Sara Wijburg, Senna van Iersel, Siméon de Bruijn, Stijn Andeweg, Sjoerd Wierenga, Susan Lanooij, Sylvia Keijser, Tara Smit, Don Klinkenberg, Jantien Backer, Pieter de Boer, Scott McDonald, Amber Maxwell, Annabel Niessen, Brechje de Gier, Danytza Berry, Daphne van Wees, Dimphey van Meijeren, Eric R.A. Vos, Frederika Dijkstra, Jeanet Kemmeren, Kylie Ainslie, Marit Middeldorp, Marjolein Kooijman, Mirjam Knol, Shahabeh Abbas Zadeh, Timor Faber, Albert Jan van Hoek, Eveline Geubbels, Hester de Melker, Jacco Wallinga, Rianne van Gageldonk-Lafeber, Susan Hahne, Susan van den Hof

**Affiliations:** 1 Center for Infectious Disease Control, National Institute for Public Health and the Environment (RIVM), Bilthoven, the Netherlands; 2 GGD Brabant Zuidoost, Eindhoven, the Netherlands; 3 The members of the group are listed under Investigators

**Keywords:** SARS-CoV-2, transmission, household study, COVID-19, vaccine effectiveness

## Abstract

Several studies report high effectiveness of COVID-19 vaccines against SARS-CoV-2 infection and severe disease, however an important knowledge gap is the vaccine effectiveness against transmission (VET). We present estimates of the VET to household and other close contacts in the Netherlands, from February to May 2021, using contact monitoring data. The secondary attack rate among household contacts was lower for fully vaccinated than unvaccinated index cases (11% vs 31%), with an adjusted VET of 71% (95% confidence interval: 63–77).

An important question when making prognoses of the pandemic in the near future and of the need on non-pharmaceutical control measures is to what extent the vaccines reduce the likelihood of transmission from infected vaccinees. Based on routine contact monitoring data, we here estimate the vaccine effectiveness against transmission (VET) and the vaccine effectiveness against infection (VE) among household and other close contacts of confirmed cases of severe acute respiratory syndrome coronavirus 2 (SARS-CoV-2) infection in the Netherlands, between 1 February and 27 May 2021. The Alpha variant (Phylogenetic Assignment of Named Global Outbreak (Pango) lineage designation B.1.1.7) was the dominant variant in the area at that time.

## Source and contact tracing

In the Netherlands, people are encouraged to undergo SARS-CoV-2 testing free of charge when experiencing symptoms or after contact with a confirmed case [1]. Infections confirmed by PCR, loop mediated isothermal amplification (LAMP) or antigen test are notified to the regional Municipal Health Services (MHS), who perform source and contact tracing and contact monitoring [2]. During our study period, household members and other close contacts of confirmed cases needed to quarantine for 10 days post exposure. All close contacts of a confirmed case were encouraged to get tested as soon as possible after exposure. In addition, a test was recommended on the 5th day after last exposure. If negative, contacts could end quarantine. We obtained a pseudonymised minimal contact monitoring dataset from all MHS. Additional data on index cases and contacts who tested positive, including the vaccine received and date of symptom onset, was extracted from the national infectious disease notification registry. Of note, a contact becomes an index case when testing positive, so our study could include some persons both as contact and as index case. 

## Vaccination status

The time since vaccination of the index case was based on the number of days between vaccination and a date used for statistics (DUFS), which was either the reported date of symptom onset or, if that was unknown, the date of positive test result minus 2 days. For vaccinated contacts, time since vaccination was calculated as the number of days between vaccination and the date of first exposure of the contact to the index case within the infectious period of the index, which is defined during source and contact tracing as 2 days before symptom onset or 2 days before test. Partly vaccinated was defined as having received the first dose of a two-dose coronavirus disease (COVID-19) vaccine, with a time since vaccination of at least 14 days. Fully vaccinated was defined as having completed a two-dose schedule with a time since vaccination of at least 7 days, or the one-dose Janssen (Ad26.COV2-S (recombinant), Janssen-Cilag International NV, Beerse, Belgium) schedule with a time since vaccination of at least 14 days. We included only index cases aged 18 years or older because children were not eligible for vaccination at the time. Contacts aged 0–17 years were included in the VET analyses, but not in the VE analyses. In order to exclude co-primary cases, the household contacts of an index were excluded if the most likely setting of infection of the index was ‘at home’ according to the source tracing interview (excluding 44,676 contacts (15%)). Further, only SARS-CoV-2-positive contacts with a DUFS within 1 to 14 days after the DUFS of the index case were included in the analyses, to reduce misclassification of indexes and secondary cases.

## Index cases and contacts

The final dataset contained 253,168 contacts of 113,582 index cases (5,394 persons in our study were both contact and index case). Of the index cases, 622 (0.5%) were fully vaccinated and 2,088 (1.8%) were partly vaccinated. Of the contacts, 5,397 (2.1%) were fully vaccinated and 4,411 were partly vaccinated (1.7%). Characteristics of indexes and contacts are shown in Table 1. We calculated the VET via the secondary attack rate (SAR) among close contacts of confirmed index cases: 1 − (SAR_vaccinated index_/SAR_unvaccinated index_) × 100% [3]. The VE among contacts was calculated as: 1 − (AR_vaccinated contacts_/AR_unvaccinated contacts_) × 100%. Both VET and VE were estimated using a binomial generalised linear model. For parameter fitting we used the generalised estimating equations approach with exchangeable correlation structure to account for clustering of contacts belonging to the same index case [4].

**Table 1:** Characteristics of COVID-19 index cases (18 years and older), by vaccination status of the index and characteristics of contacts, and by vaccination status of the contact, the Netherlands, 1 February−27 May 2021 (n = 113,582 index cases, n = 253,168 contacts)

		Vaccination status index						Vaccination status contacts					
		Unvaccinated		Partly vaccinated		Fully vaccinated		Unvaccinated		Partly vaccinated		Fully vaccinated	
		n	%^a^	n	%^a^	n	%^a^	n	%^a^	n	%^a^	n	%^a^
Total		110,872		2,088		622		243,360		4,411		5,397	
Number of contacts by type	Household	139,802	56	2,032	50	706	55	138,095	57	1,917	43	2,528	47
	Other close contacts	108,041	44	2,004	50	583	45	105,265	43	2,494	57	2,869	53
Sex	Female	56,554	51	1,325	63	472	76	121,183	50	2,689	61	4,139	77
	Male	54,318	49	763	37	150	24	120,473	50	1,684	38	1,216	23
	Unknown/ other	0	0	0	0	0	0	1,704	1	38	1	42	1
Age (years)	0–11	0	0	0	0	0	0	42,119	17	0	0	0	0
	12–17	0	0	0	0	0	0	19,770	8	0	0	0	0
	18–29	31,736	29	209	10	122	20	57,264	24	437	10	961	18
	30–49	42,142	38	347	17	179	29	54,591	22	562	13	1,102	20
	50–74	34,383	31	1,155	55	194	31	58,688	24	2,688	61	2,280	42
	≥ 75	2,611	2	377	18	127	20	4,321	2	724	16	1,054	20
	Unknown	0	0	0	0	0	0	6,607	3	0	0	0	0
Vaccine received	Vaxzevria	NA		1,144	55	35	6	NA		2,127	48	407	8
	Comirnaty	NA		890	43	530	85	NA		1,235	28	3,312	61
	Janssen	NA		0	0	21	3	NA		0	0	83	2
	Spikevax	NA		54	3	36	6	NA		86	2	247	5
	Unknown	NA		0	0	0	0	NA		963	22	1,348	25
Household composition^b^	Couple with children	12,782	15	117	8	61	14	34,603	25	126	7	286	11
	Couple without children	33,096	40	809	58	181	43	32,264	23	908	47	914	36
	Household with > two adults	21,459	26	272	19	112	27	55,408	40	648	34	1,062	42
	Other	16,056	19	197	14	68	16	15,820	11	235	12	266	11
Month of notification date of the index case	Feb	29,953	27	196	9	43	7	62,213	26	182	4	374	7
	Mar	38,573	35	435	21	143	23	88,116	36	738	17	1,571	29
	Apr	20,648	19	448	21	151	24	45,977	19	919	21	1,252	23
	May	21,698	20	1,009	48	285	46	47,054	19	2,572	58	2,200	41

COVID-19: coronavirus disease; NA: not applicable.

^a^ Column percentage.

^b^ Only presented for index cases with household contacts (n=85,210) and for household contacts (n=142,540).

## Vaccine effectiveness against transmission

The Figure shows the crude attack rates among contacts, in relation to the vaccination status of both index and contact. The SAR was 31% among household contacts of unvaccinated index cases and 11% among household contacts of fully vaccinated index cases (Table 2). Adjusting for age of the index and contact, vaccination status of the contact and month of notification date of the index case, the VET to household contacts after full vaccination was 71% (95% confidence interval (CI): 63 to 77). The VET to other close contacts was much lower (22%; 95% CI: −5 to 43), probably because of the larger risk of the contact being infected through another source (i.e. misclassification of the index case). Stratified by vaccine received by the index case, VET values were estimated at 58% for Vaxzevria (ChAdOx1-S; AstraZeneca, Cambridge, United Kingdom), 70% for Comirnaty (BNT162b2; BioNTech/Pfizer, Mainz, Germany/New York, United States (US)), 88% for Spikevax (mRNA-1273, Moderna, Cambridge, US) and 77% for the Janssen vaccine. For all vaccines with a two-dose schedule, the adjusted VET (aVET) after one dose was considerably lower than after two doses: 15% for Vaxzevria, 26% for Comirnaty and 51% for Spikevax (Table 2).

**Figure:**
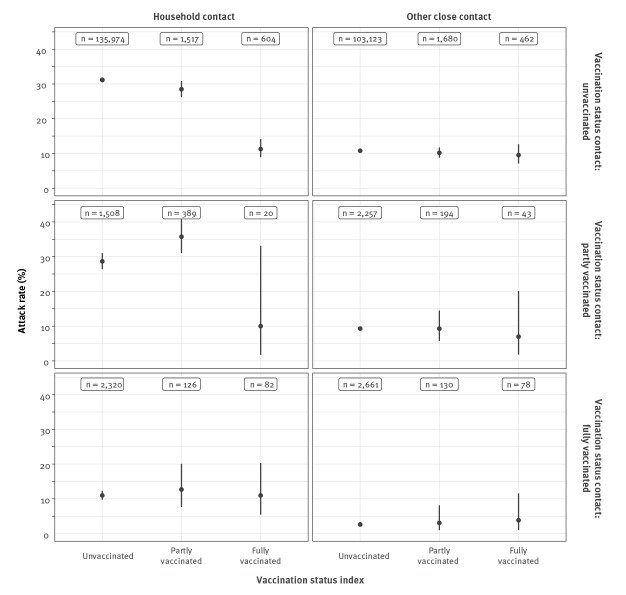
Crude attack rate of SARS-CoV-2 among contacts, by vaccination status of the index (left to right) and vaccination status of the contact (top to bottom), the Netherlands, 1 February−27 May 2021 (n = 113,582 index cases, n = 253,168 contacts)

SARS-CoV-2: severe acute respiratory syndrome coronavirus 2.

The n subtotals in the panels indicate the number of unvaccinated/partly vaccinated/fully vaccinated household and other close contacts of unvaccinated/partly vaccinated/fully vaccinated index cases. For example, n=604 in the upper left panel denotes that fully vaccinated index cases had 604 unvaccinated household contacts.

**Table 2:** Secondary attack rate of SARS-CoV-2 by vaccination status of the index case (≥ 18 years) and vaccine effectiveness against transmission^a^, crude and adjusted for age group of the index case^b^ and contact^c^ and for vaccination status^d^ of contacts and month of notification date of the index case, the Netherlands, 1 February−27 May 2021 (n = 113,582 index cases, n = 253,168 contacts)

Analysis	Unvaccinated index - SAR			Partly vaccinated index - SAR			Partly vaccinated index - crude VET (95% CI)	Partly vaccinated index - adjusted VET (95% CI)	Fully vaccinated index - SAR			Fully vaccinated index - crude VET (95% CI)	Fully vaccinated index - adjusted VET (95% CI)
	Positive	Total	%	Positive	Total	%			Positive	Total	%		
Household contacts	43,069	139,802	31	587	2,032	29	9 (−1 to 17)	21 (12 to 28)	79	706	11	72 (65 to 78)	71 (63 to 77)
Household contacts - unvaccinated only	42,382	135,974	31	432	1,517	28	12 (1 to 21)	23 (14 to 32)	68	604	11	73 (64 to 79)	73 (65 to 79)
Other close contacts	11,395	108,041	11	193	2,004	10	11 (−4 to 23)	22 (9 to 33)	50	583	9	22 (−5 to 42)	22 (−5 to 43)
Other close contacts - unvaccinated only	11,115	103,123	11	171	1,680	10	7 (−9 to 21)	22 (8 to 34)	44	462	10	14 (−18 to 37)	24 (−5 to 45)
Household contacts - Vaxzevria	43,069	139,802	31	364	1,306	28	13 (1 to 23)	15 (4 to 26)	5	39	NP	67 (16 to 87)	58 (−12 to 84)
Household contacts - Comirnaty	43,069	139,802	31	211	663	32	−4 (−22 to 12)	26 (12 to 37)	70	596	12	71 (62 to 77)	70 (61 to 77)
Household contacts - Spikevax	43,069	139,802	31	12	63	NP	46 (−3 to 71)	51 (8 to 74)	2	40	NP	88 (51 to 97)	88 (50 to 97)
Household contacts - Janssen	43,069	139,802	31	NA			NA	NA	2	31	NP	85 (37 to 96)	77 (6 to 94)

CI: confidence interval; GEE: generalised estimating equations; NA: not applicable; NP: not presented; SAR: secondary attack rate; SARS-CoV-2: severe acute respiratory syndrome coronavirus 2; VET: vaccine effectiveness against transmission.

^a^ VET estimated by GEE with exchangeable correlation structure.

^b^ Age groups index cases: 18–29, 30–49, 50–74, ≥75 years.

^c^ Age groups contacts: 0–17, 18–59, ≥60 years.

^d^ Vaccination status of contacts: unvaccinated, partly or fully vaccinated.

Partly vaccinated was defined as having received the first dose of a two-dose schedule at least 14 days before onset of symptoms. Fully vaccinated was defined as having completed a two-dose schedule at least 7 days or the one-dose Janssen schedule at least 14 days before symptom onset. Only percentages based on denominators above 100 are presented, please note the VET estimates based on smaller numbers are less precise.

## Vaccine effectiveness among contacts

The adjusted VE (aVE) for fully vaccinated household contacts of confirmed cases was estimated at 75% (95% CI: 72 to 78) and for fully vaccinated other close contacts at 79% (95% CI: 74 to 83) (Table 3). Stratified by the vaccine received by the contact, aVE was 87% for Vaxzevria, 65% for Comirnaty, 91% for Spikevax and 12% for Janssen’s vaccine. Note that the estimate for the Janssen vaccine was based on only 44 vaccinated contacts, with a median time since vaccination of 21 days. The proportion of vaccinated contacts with unknown vaccine manufacturer was large (Table 1), which reduces the power of the analyses stratified by vaccine.

**Table 3:** Attack rate among close contacts (≥ 18 years) of confirmed SARS-CoV-2 infected index cases, and vaccine effectiveness against infection^a^, crude and adjusted for age group of the index case^b^ and contact^c^ and for vaccination status of index^d^, and month of notification date of the index case, the Netherlands, 1 February−27 May 2021 (n =184,672 contacts)

Analysis	Unvaccinated contacts - AR			Partly vaccinated contacts - AR			Partly vaccinated contacts - crude VE (95% CI)	Partly vaccinated contacts - adjusted VE (95% CI)	Fully vaccinated contacts - AR			Fully vaccinated contacts - crude VE (95% CI)	Fully vaccinated contacts - adjusted VE (95% CI)
	Positive	Total	%	Positive	Total	%			Positive	Total	%		
Household contacts	32,086	91,528	35	573	1,917	30	21 (13 to 28)	23 (14 to 30)	280	2,528	11	76 (73 to 79)	75 (72 to 78)
Household contacts - only unvaccinated indexes	31,694	90,066	35	432	1,508	29	25 (17 to 33)	26 (17 to 34)	255	2,320	11	77 (74 to 79)	76 (73 to 79)
Other close contacts	9,883	83,336	12	231	2,494	9	24 (13 to 34)	28 (17 to 37)	77	2,869	3	79 (74 to 83)	79 (74 to 83)
Other close contacts - only unvaccinated indexes	9,699	81,666	12	210	2,257	9	24 (12 to 34)	27 (15 to 37)	70	2,661	3	79 (74 to 84)	80 (74 to 84)
Household contacts - Vaxzevria	32,086	91,528	35	356	1,052	34	5 (−8 to 16)	2 (−11 to 14)	11	186	6	88 (78 to 93)	87 (77 to 93)
Household contacts - Comirnaty	32,086	91,528	35	194	467	42	−30 (−56 to −8)	−18 (−43 to 2)	242	1,605	15	67 (62 to 71)	65 (60 to 70)
Household contacts - Spikevax	32,086	91,528	35	11	48	NP	45 (−5 to 71)	33 (−27 to 64)	4	121	3	93 (83 to 97)	91 (79 to 97)
Household contacts - Janssen	32,086	91,528	35	NA			NA	NA	11	44	NP	37 (−21 to 67)	12 (−71 to 54)

AR: attack rate; CI: confidence interval; GEE: generalised estimating equations; NA: not applicable; NP: not presented; SARS-CoV-2: severe acute respiratory syndrome coronavirus 2; VE: vaccine effectiveness.

^a^ VE estimated by GEE with exchangeable correlation structure.

^b^ Age groups index cases: 18–29, 30–49, 50–74, ≥75 years.

^c^ Age groups contacts: 0–17, 18–59, ≥60 years.

^d^ Vaccination status of contacts: unvaccinated, partly or fully vaccinated.

Only contacts 18 years and older were included. Partly vaccinated was defined as having received the first dose of a two-dose schedule at least 14 days before onset of symptoms. Fully vaccinated was defined as having completed a two-dose schedule at least 7 days or the one-dose Janssen schedule at least 14 days before symptom onset. Only percentages based on denominators above 100 are presented, please note the VE estimates based on smaller numbers are less precise.

## Discussion

We estimated a VET of 71% among household contacts of fully vaccinated index cases. Harris et al. found a VET of 40–50% for unvaccinated households contacts [5]. That study mostly included partly vaccinated index cases. A study among household contacts of healthcare workers by Shah et al. found a 30% (after the first dose) and 64% (after the second dose) reduction in the risk of confirmed SARS-CoV-2 infection among household members of vaccinated healthcare workers, however these healthcare workers were not confirmed as index cases [6].

Martínez-Baz et al. recently estimated VE among 20,961 close contacts of confirmed cases in Spain [7]. In that study, the VE for two doses of Comirnaty was 65% (95% CI: 56 to 73) against infection, which is in agreement with our finding of 65% VE for this vaccine. These estimates are lower than VE from other observational post-marketing studies. Possibly, the VE against infection is lower when there is high and prolonged exposure to SARS-CoV-2, which is likely for household contacts of confirmed cases [8]. Of note, Martínez-Baz et al. showed an aVE for Comirnaty of 94% (95% CI: 60 to 99) against hospitalisation among close contacts of confirmed cases.

As our study used data not primarily collected for research purposes, it has some important limitations. Our data do not contain information on negative tests among contacts, therefore we do not know if contacts did not get infected or did not seek testing. However, it is likely that close contacts were tested regardless of vaccination status, as the quarantine period for close contacts at the time was reduced from 10 to 5 days when tested negative on day 5 after exposure. For contacts who tested positive, data on vaccination status were more complete because missing data could be supplemented from the notifications. We explored whether this differential completeness influenced our results by excluding all index cases with any household contact with unknown vaccination status or date of vaccination, and the results were the same (data not shown).

Although we tried to minimise misclassification of indexes and contacts by excluding index cases infected at home and contacts with symptom onset before or at the same time as the index, it is plausible that in some instances, the transmission route was reversed or transmission occurred though another source (especially for non-household contacts). If some contacts of vaccinated index cases were infected through other sources, our VET is an underestimation. In addition, we do not have reliable data on the symptoms of the included index cases. Because our analysis on household contacts was restricted to notified index cases not infected at home, probably most of these index cases sought testing because they had symptoms. Symptomatic cases may have been misclassified as index cases in a household, where in reality an asymptomatic household member was the source of transmission to the supposed index case and a third household member. If vaccinees are more likely to be asymptomatic, this source of misclassification may result in an overestimation of the VET.

As the Alpha variant of SARS-CoV-2 dominated during the study period, an important question is to what extent these VET and VE estimates hold in the context of the Delta variant (Pango lineage designation B.1.617.2) which is now dominant in the Netherlands. Also, further research is needed to determine whether the observed differences between the different vaccines are due to the small sample size or have real public health relevance. We will prospectively monitor both VET and VE among household contacts over the next months to address these questions.

## Conclusion

Our study showed that the COVID-19 vaccines not only protect the vaccinee against SARS-CoV-2 infection, but also offer protection against transmission to close contacts after completing the full schedule. This finding underscores the importance of full vaccination of close contacts of vulnerable persons.
